# Além da Balança: A Obesidade como Fator Estrutural da Hipertensão Refratária

**DOI:** 10.36660/abc.20260139

**Published:** 2026-04-01

**Authors:** Eduardo Tibirica

**Affiliations:** 1 Instituto Nacional de Cardiologia Rio de Janeiro RJ Brasil Instituto Nacional de Cardiologia, Rio de Janeiro, RJ – Brasil

**Keywords:** Hipertensão, Pressão Arterial, Obesidade

A hipertensão arterial continua sendo um desafio global, mas seus fenótipos extremos — Hipertensão Resistente (HAR) e Hipertensão Refratária (HARf) — representam a principal causa de risco cardiovascular. Enquanto a HAR envolve pressão arterial (PA) descontrolada apesar do uso de três classes de medicamentos, a HARf é sua manifestação mais grave, definida pela falha no tratamento com cinco ou mais medicamentos.^1^ No Brasil, a HAR é altamente prevalente; o estudo ELSA-Brasil relatou uma prevalência de 11% entre pacientes tratados, com maiores riscos para indivíduos idosos, negros e obesos.^2^

Expandindo esse panorama, Costa et al.^3^ fornecem uma análise oportuna de uma coorte de alta complexidade. Suas descobertas revelam uma correlação notável entre o índice de massa corporal e a carga farmacológica (r = 0,45; p < 0,01). Essa relação dose-resposta sugere que, em indivíduos obesos, o "efeito teto" da terapia convencional é atingido mais rapidamente, exigindo uma intensificação da polifarmácia. Além disso, ao se concentrarem em uma coorte composta predominantemente por mulheres afrodescendentes com mais de 60 anos de idade, os autores abordam uma lacuna crítica em relação a grupos vulneráveis frequentemente sub-representados em pesquisas clínicas.

A relação entre adiposidade e falha terapêutica envolve uma interação complexa de mecanismos neuro-hormonais e estruturais. A gordura visceral aumenta a pressão intra-abdominal e causa compressão física dos rins, levando à ativação crônica do sistema renina-angiotensina-aldosterona.^4^ Simultaneamente, a hiperleptinemia leva à hiperatividade simpática,^5^ contribuindo ainda mais para a manutenção de níveis elevados de PA.

Contudo, do ponto de vista da microcirculação, o impacto da obesidade é ainda mais profundo e insidioso. A inflamação crônica de baixo grau e a lipotoxicidade induzidas pela obesidade promovem estresse oxidativo sistêmico,^6^ o que prejudica significativamente a biodisponibilidade do óxido nítrico e desencadeia alterações microvasculares, incluindo rarefação capilar funcional e remodelação arteriolar interna.^7-9^ Essa disfunção endotelial e a consequente rigidez microvascular reduzem drasticamente a capacidade de resposta do leito vascular a vasodilatadores endógenos e farmacológicos.^10^ Consequentemente, se estabelece uma "barreira estrutural", que explica eficazmente a natureza intratável da PA e a fraca resposta à polifarmácia intensiva observada no grupo refratário estudado por Costa et al.^3^

Além disso, as evidências fornecidas por Costa et al.^3^ sugerem que a polifarmácia tradicional frequentemente atinge um "efeito teto" em pacientes obesos refratários. Nesse cenário, o tratamento da adiposidade se torna a principal estratégia anti-hipertensiva, em vez de um objetivo secundário. Intervenções metabólicas emergentes, como agonistas do receptor de GLP-1 e cirurgia bariátrica, esta última demonstrando notável eficácia no controle da PA e na redução da medicação em casos de HAR, conforme demonstrado no estudo GATEWAY, um marco na área^11,12^, oferecem vias promissoras ao visar diretamente os gatilhos neuro-hormonais subjacentes da resistência.

Em última análise, este estudo reforça que, na luta contra a hipertensão grave, a balança é tão importante quanto o esfigmomanômetro. Atingir as metas de PA nessas populações vulneráveis e sub-representadas exige uma mudança de paradigma, passando da escalada puramente farmacológica para a restauração metabólica abrangente.
