# Supplementary material for: Além da Balança: A Obesidade como Fator Estrutural da Hipertensão Refratária
Source: Arq Bras Cardiol. 2026 Apr 1;123(2):e20260139. [Article in Portuguese] doi: 10.36660/abc.20260139 (PMC13128175; doi:10.36660/abc.20260139)
Supplement: Translation

# Translation: Beyond the Scale: Obesity as a Structural Driver of Refractory Hypertension

**DOI:** 10.36660/abc.20260139i

**Authors:** Eduardo Tibirica

**Affiliations:** 1 Rio de Janeir RJ Brazil Instituto Nacional de Cardiologia, Rio de Janeiro, RJ – Brazil

**Keywords:** Hypertension, Arterial Pressure, Obesity

Arterial hypertension remains a global challenge, yet its extreme phenotypes—Resistant Hypertension (RAH) and Refractory (RefH) Hypertension—represent the forefront of cardiovascular risk. While RAH involves uncontrolled blood pressure (BP) despite three drug classes, RefH is its most severe manifestation, defined by failure with five or more drugs.^1^ In Brazil, RAH is highly prevalent; the ELSA-Brasil study reported an 11% prevalence among treated patients, with higher risks for older, Black, and obese individuals.^2^

Expanding this landscape, Costa et al.^3^ provide a timely analysis of a high-complexity cohort. Their findings reveal a striking correlation between body mass index and pharmacological burden (r = 0.45; p < 0.01). This "dose-response" relationship suggests that in obese individuals, the "ceiling effect" of conventional therapy is reached sooner, necessitating an escalation of polypharmacy. Furthermore, by focusing on a cohort predominantly composed of Afro-descendant women over 60 years of age, the authors address a critical gap regarding vulnerable groups often underrepresented in clinical research.

The link between adiposity and therapeutic failure involves a complex interplay of neuro-hormonal and structural mechanisms. Visceral fat increases intra-abdominal pressure and causes physical renal compression, leading to the chronic activation of the renin-angiotensin-aldosterone system.^4^ Simultaneously, hyperleptinemia drives sympathetic overactivity,^5^ further sustaining high BP levels.

However, from a microcirculatory perspective, the impact of obesity is even more profound and insidious. Obesity-induced chronic low-grade inflammation and lipotoxicity foster systemic oxidative stress,^6^ which significantly impairs nitric oxide bioavailability and triggers microvascular alterations, including functional capillary rarefaction and inward arteriolar remodeling.^7-9^ This endothelial dysfunction and subsequent microvascular stiffness drastically reduce the vascular bed's responsiveness to both endogenous and pharmacological vasodilators.^10^ Consequently, a "structural barrier" is established, effectively explaining the intractable nature of BP and the poor response to heavy polypharmacy observed in the refractory group studied by Costa et al.^3^

Moreover, the evidence provided by Costa et al.^3^ suggest that traditional polypharmacy often reaches a "ceiling effect" in obese refractory patients. In this scenario, addressing adiposity becomes the primary antihypertensive strategy rather than a secondary goal. Emerging metabolic interventions, such as GLP-1 receptor agonists and bariatric surgery—the latter having demonstrated remarkable efficacy in achieving BP control and medication reduction in resistant hypertension, as shown in the landmark GATEWAY trial^11,12^, offer promising pathways by directly targeting the underlying neuro-hormonal triggers of resistance.

Ultimately, this study reinforces that in the battle against severe hypertension, the weighing scale is as critical as the sphygmomanometer. Achieving BP targets in these vulnerable and underrepresented populations requires a paradigm shift, moving from purely pharmacological escalation toward comprehensive metabolic restoration.
